# Association Between Pre-Transfusion Systemic Inflammatory Response Syndrome and Transfusion-Associated Adverse Events in Dogs: A Retrospective Study

**DOI:** 10.3390/ani16203287

**Published:** 2026-10-18

**Authors:** Kyung-Mi Lee, Yi-Kyeong Jeong, Joon-Yong Jung, Hyun-Jeong Sung, Ji Heui Sohn, Ju-Hyun An

**Affiliations:** 1 Department of Veterinary Emergency and Critical Care Medicine, Institute of Veterinary Science, College of Veterinary Medicine, Kangwon National University, Chuncheon-si 24341, Republic of Korea; lkm0026@gmail.com; 2 VIP Animal Medical Center, 7, Samseong-ro 133-gil, Gangnam-gu, Seoul 06068, Republic of Korea; lauren100412@gmail.com (Y.-K.J.); calmtown@naver.com (J.-Y.J.); sunghj0828@naver.com (H.-J.S.); wlgml924@naver.com (J.H.S.)

**Keywords:** canine, blood transfusion, transfusion-associated adverse event, systemic inflammatory response syndrome

## Abstract

Blood transfusion is an important supportive therapy in critically ill dogs, but transfusion reactions (TRs) remain a clinically relevant complication. This retrospective study evaluated whether pre-transfusion systemic inflammatory response syndrome (SIRS) was associated with transfusion-associated adverse events in dogs. Medical records of dogs receiving red-cell-containing blood products at a veterinary referral hospital in the Republic of Korea between December 2023 and May 2026 were reviewed. Among 146 transfusion episodes from 100 dogs, 142 episodes with evaluable primary outcomes were included in the primary outcome analysis. Transfusion-associated adverse events were identified more frequently in the SIRS group than in the No SIRS group (32.7% vs. 11.4%; *p* = 0.007). In univariable logistic regression, SIRS was associated with increased odds of the primary outcome (OR, 3.78; 95% CI, 1.36–10.51; *p* = 0.011). In a patient-clustered multivariable generalized estimating equation (GEE) analysis, the adjusted OR remained elevated at 3.57, although the association did not reach statistical significance (95% CI, 0.98–12.98; *p* = 0.053). Each additional fulfilled SIRS criterion was associated with increased odds of the primary outcome (OR, 1.63; 95% CI, 1.10–2.41; *p* = 0.014). However, the association was attenuated and was not statistically significant in sensitivity analyses using stricter outcome definitions based on imputability and independent re-adjudication. These findings suggest an association between pre-transfusion SIRS and clinically suspected transfusion-associated adverse events under the broad retrospective primary outcome definition, but the results were sensitive to the level of diagnostic certainty required for outcome classification. The findings should therefore be considered hypothesis-generating and require confirmation in prospective studies using standardized reaction definitions and blinded outcome assessment.

## Simple Summary

Blood transfusions are commonly used to treat dogs with severe anemia or blood loss, but adverse transfusion reactions can occur. Dogs requiring transfusion are often critically ill and may have systemic inflammation; however, it remains unclear whether this inflammatory state is associated with transfusion-associated adverse events. In this retrospective study, transfusion-associated adverse events were identified more frequently in transfusion episodes with pre-transfusion systemic inflammatory response syndrome than in those without it, and a greater number of fulfilled syndrome criteria were associated with higher odds of the primary outcome. However, critically ill dogs can worsen for several reasons, and retrospective medical records may not provide enough evidence to determine with high certainty whether an adverse event was caused by transfusion. The association became weaker and was not statistically significant when stricter outcome definitions requiring greater certainty or independent reassessment were used. These findings should therefore be considered hypothesis-generating and require confirmation in prospective studies using standardized reaction assessment.

## 1. Introduction

Blood transfusion is a life-saving therapeutic intervention in veterinary medicine, particularly for patients with severe anemia, hemorrhage, hemolysis of various etiologies, or impaired erythropoiesis [1,2]. However, it carries inherent risks, including the development of transfusion reactions (TRs) that can range from mild, self-limiting signs to life-threatening complications [3,4]. Several risk factors for TRs have been identified in dogs, including patient-related factors such as underlying immune-mediated diseases and product-related factors such as prolonged storage times and higher doses of packed red blood cells (pRBCs) [5,6].

Systemic inflammatory response syndrome (SIRS) is a nonspecific systemic inflammatory response to various infectious or non-infectious insults [7,8]. In veterinary medicine, SIRS is commonly identified using established canine SIRS criteria. According to these criteria, dogs are considered to have SIRS when at least two of four criteria are present: abnormal body temperature, tachycardia, tachypnea, and white blood cell abnormalities [9].

Blood transfusion itself may provide an additional inflammatory stimulus [10,11]. Therefore, dogs with pre-existing systemic inflammation could plausibly be more susceptible to TRs, consistent with a “two-hit” framework in which an existing inflammatory state primes the host response to a subsequent transfusion-related stimulus [10,12]. However, evaluating this relationship is challenging because several clinical abnormalities used to identify SIRS overlap with manifestations of TRs [3,9]. Consequently, retrospective classification of TRs can be challenging, as clinical deterioration caused by the underlying disease can mimic a transfusion reaction [13,14]. This diagnostic overlap complicates assessment of the relationship between pre-transfusion SIRS and subsequent TRs in dogs. 

Given this uncertainty, the primary objective of this study was to compare the occurrence of transfusion-associated adverse events between transfusion episodes in dogs with and without pre-transfusion SIRS. Secondary objectives were to evaluate other potential risk factors for the primary outcome and to examine the association between the number of fulfilled SIRS criteria and the primary outcome. To assess the robustness of the observed association to potential misclassification of primary outcome status, we performed independent blinded re-adjudication and sensitivity analyses. We hypothesized that transfusion episodes in dogs with pre-transfusion SIRS would have a higher observed frequency of transfusion-associated adverse events than those in dogs without SIRS.

## 2. Materials and Methods

### 2.1. Study Design and Population

This retrospective observational study reviewed the medical records of client-owned dogs that received red-cell-containing blood products at VIP Animal Medical Center Cheongdam branch (Seoul, Republic of Korea) between December 2023 and May 2026. The study population was restricted a priori to stored whole blood and pRBC transfusions; fresh frozen plasma and other non-red-cell blood products were outside the study scope. 

A transfusion episode was defined as a single planned course of transfusion. Multiple syringe aliquots from the same blood unit administered sequentially as part of the same planned transfusion were considered one episode. If transfusion was terminated early because of a suspected adverse event and was not resumed, the initiated transfusion was still counted as one episode. A later, separately planned transfusion course was treated as a new episode, including repeated transfusions occurring during the same hospitalization or during a subsequent admission, even when the remaining portion of the same blood unit was used. Dogs could therefore contribute more than one episode, and patient identification was retained for clustered analyses. Because pre-transfusion SIRS reflected the dog’s clinical status at the time of each transfusion and could differ between episodes in the same dog, the transfusion episode was used as the primary unit of analysis.

Pre-transfusion SIRS was assessed using all four SIRS criteria, with data for all four criteria required for classification. Primary outcome status was determined from available post-transfusion clinical information. Episodes with insufficient information to determine primary outcome status were considered non-evaluable rather than as not having met the primary outcome.

### 2.2. Data Collection and Definition of Pre-Transfusion SIRS

Demographic, clinical, laboratory, and transfusion-related data were retrospectively extracted from the medical records, including transfusion-monitoring sheets. Variables included age, breed, sex and neuter status, body weight, systolic blood pressure (SBP), C-reactive protein (CRP), hematocrit (HCT), platelet count, body temperature, heart rate, respiratory rate, white blood cell (WBC) count, underlying diagnoses, blood product type, actual transfusion volume (mL/kg), actual transfusion duration, and documented previous transfusion exposure. Underlying diseases were categorized as immune-mediated, infectious/inflammatory, neoplastic, gastrointestinal, hepatobiliary, renal, hemorrhagic, endocrine/metabolic, other causes of anemia, or other diseases.

Temperature, heart rate, and respiratory rate used for SIRS classification were the values documented immediately before transfusion on the transfusion-monitoring sheet. Temperature was measured rectally. Systolic blood pressure was measured using Doppler ultrasonography or invasive arterial blood pressure monitoring when an arterial catheter was available. The WBC count was obtained from the same-day CBC performed before transfusion and closest to transfusion initiation. CBCs were performed using a ProCyte Dx Hematology Analyzer (IDEXX Laboratories, Westbrook, ME, USA). CRP was measured in mg/L using the Vcheck canine CRP assay (Bionote, Hwaseong-si, Republic of Korea); the institutional reference interval was 0–20 mg/L.

Pre-transfusion SIRS was defined as fulfillment of at least two of the following four criteria: rectal temperature < 38.1 °C or >39.2 °C; heart rate > 120 beats/min; respiratory rate > 20 breaths/min; and WBC count < 6000/µL or >16,000/µL [9]. Quantitative band-neutrophil percentages were not consistently available in the retrospective records; therefore, the leukocyte component of the SIRS definition was based on total WBC count, and band neutrophilia was not used as an additional criterion. Analyzer flags suggesting immature leukocytes were reviewed as supportive information only and did not alter SIRS classification. SIRS status was assigned only when data for all four components were available. Respiratory rate was analyzed as recorded; values > 20 breaths/min were considered positive for the respiratory criterion even when panting, pain, or stress was documented.

Previous transfusion exposure was considered present when at least one transfusion before the index episode was documented in available medical or referral records or in the owner-reported medical history. The exact lifetime number of prior transfusions was not consistently ascertainable for dogs transfused outside the study hospital. No imputation was performed for missing data; analyses involving CRP were restricted to episodes in which CRP had been measured.

### 2.3. Blood Products, Compatibility Testing, and Transfusion Protocol

All whole-blood units used in the study were refrigerated stored blood; no fresh whole-blood transfusions were included. Blood was collected into CPDA-containing bags and discarded 21 days after collection. The exact storage age of the individual unit administered in each episode could not be consistently determined retrospectively. Commercially supplied whole blood predominated. During the study period, commercial pRBC units were not available to the institution; pRBC units were therefore prepared in-house when needed by centrifugation of donated whole blood followed by plasma removal. Packed cells remained in the original CPDA collection bag and were stored under the same refrigerated conditions, with a maximum storage period of 21 days from collection. The predominance of whole blood in the cohort primarily reflected local product availability rather than a clinical preference for whole blood over pRBCs.

Most whole-blood units were obtained from a commercial veterinary blood bank. Detailed donor-screening procedures and individual donor test results for commercially supplied units were not available in the retrospective records. In-house donors were clinically healthy dogs ≤ 6 years of age with no history of previous transfusion under institutional donor-selection criteria. In-house donor assessment included CBC, serum biochemistry, venous blood gas analysis, and PCR screening for tick-borne pathogens using a commercial diagnostic panel (PopAniLab, Namyangju-si, Republic of Korea) [15,16].

Recipient DEA 1 type was determined with a commercial blood-typing kit when a reliable previous typing result was unavailable; previously confirmed DEA 1 results were used for subsequent transfusions. DEA 1-negative recipients received DEA 1-negative blood, whereas DEA 1-positive recipients could receive DEA 1-positive or DEA 1-negative blood. Major and minor crossmatches were performed by a conventional tube method whenever a new donor blood unit was introduced [17]. Within a single planned transfusion episode, the prescribed volume could be divided into multiple syringe aliquots from the same blood unit, and crossmatching was not repeated for individual aliquots. A remaining portion of the same blood unit could also be used for a later, separately planned transfusion episode in the same dog. In such cases, crossmatching was not repeated when the same unit was reused within 4 days of the preceding transfusion; crossmatching was repeated when >4 days had elapsed. Compatibility was assessed macroscopically and microscopically for agglutination, with rouleaux formation distinguished from true agglutination. Overt hemolysis, when observed, also prompted selection and crossmatching of another unit. Donor identity and the number of distinct donor exposures could not be consistently determined for all episodes and were not analyzed.

A standard in-line blood administration filter was used for all transfusions; leukoreduction was not performed. All episodes received premedication. Dexamethasone (0.1 mg/kg IV) and chlorpheniramine (0.2 mg/kg SC) were the principal agents and were most commonly administered together, although some dogs received only one of these agents at the attending veterinarian’s discretion. Transfusions were initiated at 0.5–1 mL/kg/h. In the absence of adverse changes, the subsequent infusion rate was individualized according to underlying disease, volume status, and illness severity; no fixed institutional maximum infusion rate was used.

Temperature, heart rate, respiratory rate, and SBP were monitored at 5, 10, 15, and 30 min after transfusion initiation and hourly thereafter during transfusion. New clinical abnormalities prompted interruption of transfusion and reassessment, including diagnostic imaging when clinically indicated. In the study episodes in which a reaction was suspected and transfusion was interrupted, that transfusion was not resumed. If additional transfusion support was later required, a new blood unit was used in a separately planned episode. Post-transfusion monitoring frequency and duration were individualized to clinical condition; no standardized post-transfusion monitoring schedule was used.

For assessment of later reactions, all available subsequent hospital records were reviewed, including clinical signs, physical examination findings, laboratory results, and diagnostic imaging when performed. When a subsequent hospital visit was unavailable, documented telephone follow-up with the owner was reviewed when present. No standardized minimum follow-up interval was prespecified. Dogs discharged immediately after transfusion without subsequent clinical records or documented owner follow-up could not be adequately assessed for a post-transfusion reaction.

### 2.4. Definition and Classification of Transfusion-Associated Adverse Events

The primary outcome was the occurrence of a retrospectively identified transfusion-associated adverse event. Acute events were defined as events beginning during transfusion or within 24 h after completion, whereas events beginning after 24 h were considered delayed [3]. Primary outcome classification was performed retrospectively by the first author through review of transfusion-monitoring records, clinical signs, vital signs, laboratory findings, and diagnostic imaging when available. Documentation by the attending veterinarian that a TR had occurred was not sufficient by itself to define the outcome. The first author was not blinded to pre-transfusion SIRS status. Classification was guided by the 2021 AVHTM Transfusion Reaction Small Animal Consensus Statement (TRACS) definitions where applicable [3]. Events not encompassed by a TRACS-defined category were considered study-specific non-TRACS transfusion-associated adverse events when they developed in temporal association with transfusion, were newly observed relative to the pre-transfusion state, and no more plausible alternative explanation was identified from the available clinical information. These events were included in the primary outcome. Formal imputability and severity grades were not assigned during the original review. Event categories described below were limited to those that could be meaningfully assessed from the available retrospective records.

Operational criteria were applied relative to each dog’s pre-transfusion status whenever possible. Suspected acute hemolytic transfusion reaction (AHTR) required evidence of new or worsening hemolysis within 24 h, operationalized as new or worsening hyperbilirubinemia, defined by a baseline-relative increase in total bilirubin with the post-transfusion concentration above the institutional reference interval, together with a post-transfusion HCT response below the calculated expected value. Additional findings such as hemoglobinemia, hemoglobinuria, or bilirubinuria were considered supportive when documented but were not required because they were not consistently available. The target HCT was individualized by the attending veterinarian. Transfusion dosing used the equation: transfusion volume (mL) = body weight (kg) × 90 mL/kg × [(target HCT − recipient HCT)/donor HCT] [18]. Donor HCT was measured directly from the blood unit before transfusion. For assessment of the post-transfusion HCT response, the expected post-transfusion HCT was calculated from the actual administered volume as follows: expected post-transfusion HCT = pre-transfusion HCT + [(actual transfused volume × donor HCT)/(body weight × 90 mL/kg)]. Accordingly, for transfusions terminated before completion of the planned volume, the expected post-transfusion HCT was calculated using the actual administered volume.

Respiratory episodes occurring during transfusion or within 6 h after completion were retrospectively classified as suspected TRALI when the overall clinical pattern was considered more consistent with acute non-hydrostatic pulmonary injury than with circulatory overload. The assessment incorporated the onset and progression of respiratory signs, changes in thoracic radiographic findings when available, blood pressure, cardiac silhouette size on thoracic radiographs, response to diuretic therapy, and competing causes of respiratory deterioration. Because standardized pre- and post-transfusion thoracic imaging was not available in every case and definitive distinction from TACO or other causes of acute lung injury was not always possible, these episodes were classified as suspected TRALI [3,14].

Respiratory episodes occurring during transfusion or within 6 h after completion were retrospectively classified as suspected TACO when the overall clinical pattern was considered more consistent with hydrostatic volume overload. The assessment incorporated changes in respiratory status and thoracic radiographic findings, including pulmonary edema with or without pleural effusion, changes in cardiac silhouette size, blood pressure, documented fluid administration and volume status, response to diuretic therapy, and other potential causes of respiratory deterioration when available. Alternative explanations, including pneumonia, acute respiratory distress syndrome, progression of the underlying critical illness, and non-transfusion fluid administration, were considered when reviewing the records [3,14].

A study-specific suspected transfusion-associated hyperkalemic adverse event was recorded when serum potassium was within the institutional reference interval before transfusion and increased above the upper reference limit after transfusion. One such event was identified 12 h after transfusion and occurred concurrently with AHTR. Unexplained bradycardia was recorded separately as a study-specific non-TRACS suspected transfusion-associated adverse event when heart rate was within the patient’s pre-transfusion range and decreased to <60 beats/min during transfusion without administration of medication expected to cause bradycardia. Ionized calcium measurements, when available, were also reviewed when evaluating hypocalcemia as an alternative cause of bradycardia.

Febrile non-hemolytic transfusion reaction (FNHTR) was defined as a body temperature >39 °C with an increase >1 °C from the pre-transfusion value during transfusion or within 4 h after completion, in the absence of a more plausible explanation such as external warming or progression of the underlying disease [3].

Allergic transfusion reactions were considered when new compatible cutaneous, gastrointestinal, respiratory, or cardiovascular signs developed during transfusion or within 4 h after completion. Compatible cutaneous findings included erythema, urticaria, pruritus, and angioedema [3]. Tachycardia and hypotension recorded during routine transfusion monitoring were considered supportive systemic findings but were interpreted in the context of other potential causes. Anaphylaxis was considered when acute cardiovascular or respiratory compromise, such as hypotension, collapse, or respiratory distress, occurred in temporal association with transfusion, even in the absence of cutaneous or gastrointestinal signs, after consideration of other plausible causes.

Delayed hemolytic transfusion reaction (DHTR) was assessed from available follow-up records from >24 h to 28 days after transfusion. DHTR was considered when an otherwise unexplained decrease in PCV/HCT was accompanied by at least two indicators of delayed red-cell destruction [3,19]. Available CBC results, serum total bilirubin concentrations, and clinical evidence of hemolysis, including icterus, were reviewed in combination. When a direct antiglobulin test (DAT) had been performed as part of the clinical diagnostic work-up for suspected hemolysis, the available result was also reviewed. Because standardized laboratory follow-up and serologic testing were not available for all dogs, DHTR could not be definitively excluded in all episodes.

Citrate toxicity was assessed in episodes for which paired pre- and post-transfusion ionized calcium measurements were available. Changes in ionized calcium were reviewed with reference to the TRACS criteria for citrate toxicity, including the reported ionized calcium thresholds and relevant clinical context [3]. Because ionized calcium was not measured systematically in all episodes, citrate toxicity could not be definitively excluded in dogs without paired measurements.

When dogs received multiple transfusions, a suspected transfusion-associated adverse event was assigned to the most recent transfusion episode when onset occurred within the predefined temporal window for that event type.

### 2.5. Independent Re-Adjudication and Sensitivity Analysis Methods

To assess the robustness of primary outcome classification, the 37 episodes meeting the primary outcome definition of a transfusion-associated adverse event were independently re-adjudicated by a second veterinarian with clinical experience in emergency and critical care. The second reviewer was blinded to the pre-transfusion SIRS classification and the original reaction-subtype assignment. The reviewer evaluated onset timing, baseline clinical status, new clinical signs, laboratory and imaging findings, treatment response, alternative causes, reaction presence (yes/no/uncertain), reaction subtype, and imputability. Severity was not retrospectively graded.

The independent re-adjudication was designed as a sensitivity analysis to assess the diagnostic certainty of the 37 episodes meeting the primary outcome definition rather than as a complete second ascertainment of outcome status for the entire cohort. Disagreements between the original and independent classifications were not resolved by consensus because the independent classifications were intended to remain a separate blinded outcome definition for sensitivity analysis. The original classifications were retained for the primary analysis, whereas the independent classifications were used only in sensitivity analyses. Importantly, independent re-adjudication was not performed for the 105 episodes that did not meet the primary outcome definition. Therefore, potential false-negative events among these episodes could not be assessed, and the independent re-adjudication should not be interpreted as validation of the overall accuracy or false-negative rate of the primary outcome classification.

Sensitivity analyses included: (1) reclassifying both fatal non-evaluable episodes as having met the primary outcome; (2) reclassifying both fatal episodes as not having met the primary outcome; (3) excluding study-specific non-TRACS unexplained bradycardic events; (4) restricting the outcome to events assigned definite or probable transfusion imputability at independent re-adjudication [3]; (5) restricting the outcome to episodes judged to represent a TR by the independent reviewer; and (6) restricting the outcome to independently judged TRs with a TRACS-defined subtype. An additional dog-level sensitivity analysis retained only the first evaluable study-period transfusion episode for each dog to assess the influence of repeated transfusion episodes within individual dogs.

### 2.6. Statistical Analysis

Continuous variables are presented as median and interquartile range (IQR), and categorical variables as number and percentage. Continuous variables were compared between the SIRS and No SIRS groups using the Mann–Whitney U test. Categorical variables were compared using Fisher’s exact test or Pearson’s chi-square test, as appropriate. Variables used to define SIRS status were summarized descriptively but were not subjected to between-group hypothesis testing because these variables were components of the exposure definition.

Univariable logistic regression was used to evaluate associations between candidate predictors and the occurrence of transfusion-associated adverse events. For clinical interpretability, continuous predictors were rescaled to predefined increments: systolic blood pressure (SBP) per 10 mmHg, C-reactive protein (CRP) per 10 mg/L, hematocrit (HCT) per 5 percentage points, platelet count per 50 K/µL, transfusion volume per 5 mL/kg, and transfusion duration per 1 h. These increments were selected solely to facilitate clinical interpretation of the odds ratios; rescaling did not affect model fit or statistical significance.

Because individual dogs could contribute more than one transfusion episode, multivariable generalized estimating equation (GEE) logistic regression with a binomial distribution and logit link was used to account for within-dog correlation. Patient identification was specified as the clustering variable, an exchangeable working correlation structure was used, and robust standard errors were calculated.

Given the limited number of transfusion-associated adverse events, the number of covariates included in the multivariable model was intentionally restricted to maintain model parsimony and reduce the risk of overfitting and unstable estimates. Covariate selection was guided primarily by clinical relevance to the association between pre-transfusion SIRS and transfusion-associated adverse events rather than by statistical significance in univariable analyses alone. The primary model included pre-transfusion SIRS status as the exposure of interest, blood product type as a clinically relevant transfusion-related factor, and pre-transfusion platelet count as a recipient-related hematologic factor. Underlying disease categories and individual diagnoses were considered clinically relevant potential confounders but were not simultaneously incorporated into the primary model because of their heterogeneity and overlap and the limited number of outcome events. A sensitivity GEE model additionally included documented previous transfusion exposure because prior exposure to blood products could potentially influence the occurrence of subsequent transfusion-associated adverse events.

For the SIRS-criterion count analysis, the number of fulfilled SIRS criteria (0–4) was modeled as a continuous predictor to assess its association with the odds of transfusion-associated adverse events. Wilson score 95% confidence intervals were calculated for the primary outcome proportions according to the number of fulfilled SIRS criteria.

All tests were two-sided, with *p* < 0.05 considered statistically significant. *p*-values are reported to three decimal places except when *p* < 0.001. Descriptive analyses, group comparisons, and conventional logistic regression analyses were performed using GraphPad Prism version 8 (GraphPad Software, San Diego, CA, USA). GEE analyses were performed using Python version 3.13.5 with statsmodels version 0.14.6. 

## 3. Results

### 3.1. Study Population and Baseline Characteristics

**Figure 1:**
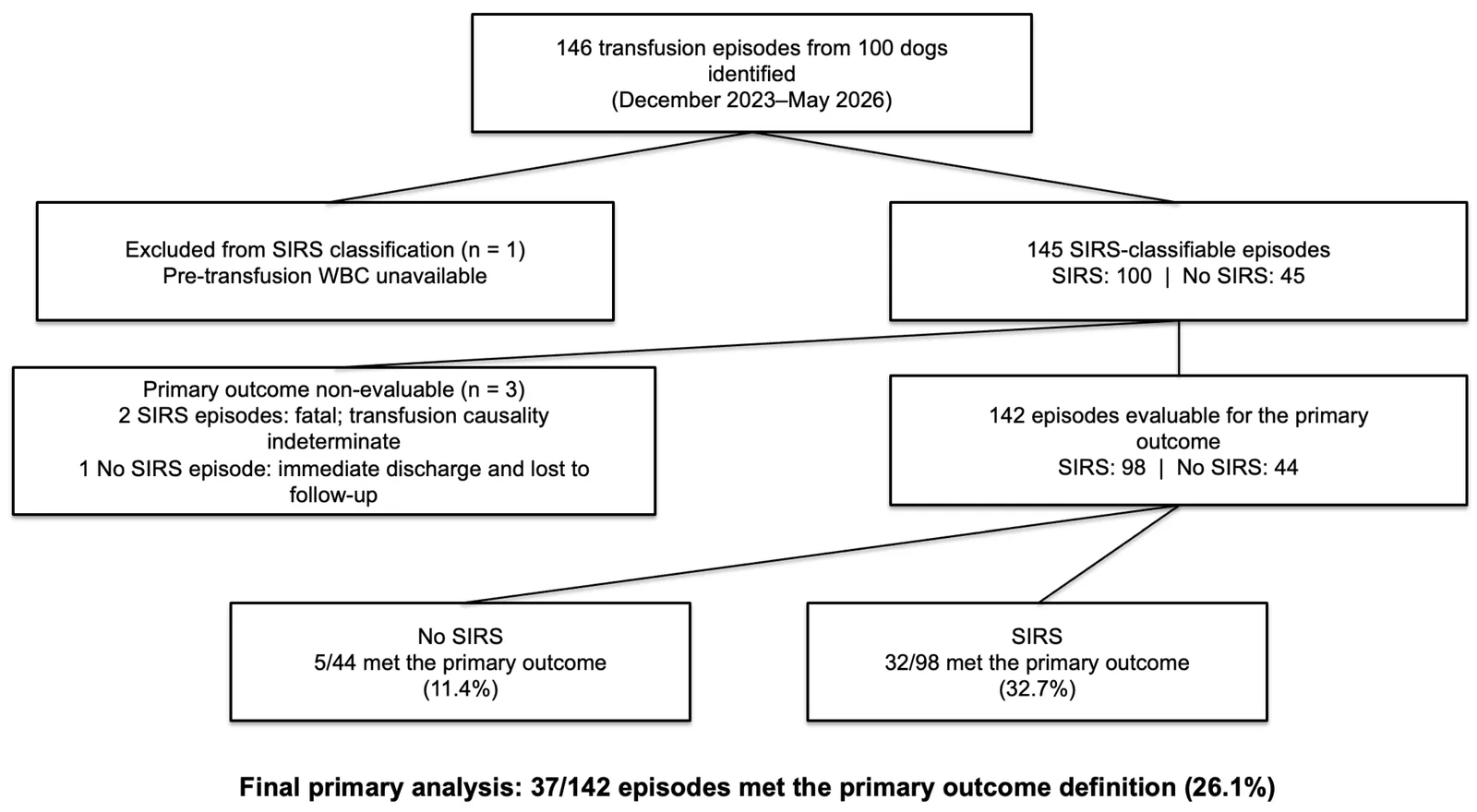
Flow diagram of transfusion episodes included in the study. A total of 146 transfusion episodes from 100 dogs were identified. One episode lacked a pre-transfusion white blood cell count and therefore could not be classified for systemic inflammatory response syndrome (SIRS), leaving 145 SIRS-classifiable episodes. Of these, three episodes were not evaluable for transfusion-associated adverse events analyses: two SIRS episodes ended in death with transfusion causality indeterminate, and one No SIRS episode was discharged immediately after transfusion and was subsequently lost to follow-up. Therefore, 142 transfusion episodes were included in the primary outcome analyses, comprising 44 No SIRS episodes and 98 SIRS episodes.

A total of 146 transfusion episodes from 100 dogs were identified during the study period. Of the 100 dogs, 66 contributed a single study-period transfusion episode and 34 contributed two or more episodes. One episode lacked a pre-transfusion WBC count and therefore could not be classified for SIRS, leaving 145 SIRS-classifiable episodes, of which 45 were classified as No SIRS and 100 as SIRS. Three SIRS-classifiable episodes were not evaluable for transfusion-associated adverse events: two SIRS episodes ended in death for which transfusion causality could not be reliably determined, and one No SIRS episode was discharged immediately after transfusion and was subsequently lost to follow-up. Therefore, 142 transfusion episodes, including 44 No SIRS and 98 SIRS episodes, were included in the primary outcome analysis. These 142 evaluable episodes represented all 100 dogs (Figure 1).

Breed and neuter status were available for all 100 dogs. At the dog level, 96/100 (96.0%) were neutered or spayed and 4/100 (4.0%) were intact. The most frequently represented breeds were Maltese (*n* = 19), Pomeranian (*n* = 14), Poodle (*n* = 12), mixed-breed (*n* = 10), Bichon Frise (*n* = 6), Chihuahua (*n* = 4), Shih Tzu (*n* = 4), and Cocker Spaniel (*n* = 4); the remaining 27 dogs represented other breeds.

Baseline characteristics according to pre-transfusion SIRS status are summarized in Table 1. Age, sex, and body weight did not differ significantly between the No SIRS and SIRS groups. SBP was significantly lower in the SIRS group than in the No SIRS group (median [IQR], 120.0 [107.2–140.0] vs. 130.0 [120.0–150.0] mmHg; *p* = 0.006). Pre-transfusion HCT was also lower in the SIRS group (16.7 [14.6–20.0]%) than in the No SIRS group (17.7 [15.2–22.9]%; *p* = 0.034).

CRP measurements were available for 90 of the 145 SIRS-classifiable episodes (27 No SIRS and 63 SIRS episodes). CRP concentrations tended to be higher in the SIRS group than in the No SIRS group (73.20 [36.37–101.75] vs. 47.63 [26.55–78.60] mg/L), although the difference did not reach statistical significance (*p* = 0.052). Similarly, pre-transfusion platelet counts tended to be lower in the SIRS group (89.5 [35.5–203.2] K/µL) than in the No SIRS group (157.0 [59.0–321.0] K/µL; *p* = 0.051). The distribution of underlying disease categories did not differ significantly between the groups (*p* = 0.067). SIRS-defining variables, including body temperature, heart rate, respiratory rate, and WBC count, were reported descriptively without between-group hypothesis testing because these variables were used to define SIRS status.

### 3.2. Transfusion Characteristics and Event Labels

Among the 142 evaluable episodes, 135 (95.1%) used stored whole blood and 7 (4.9%) used pRBCs. Documented previous transfusion exposure was present in 47/142 episodes (33.1%). Blood product type, transfusion volume, transfusion duration, and documented previous transfusion exposure did not differ significantly between the No SIRS and SIRS groups (Table 2). The predominance of whole blood reflected local blood-product availability; pRBCs were prepared in-house from donated whole blood when needed.

Transfusion-associated adverse events occurred in 37/142 episodes (26.1%): 32/98 SIRS episodes (32.7%) and 5/44 No SIRS episodes (11.4%; *p* = 0.007). All 37 identified adverse events were acute. Event labels were AHTR in 8 episodes, suspected TRALI in 8, suspected TACO in 12, a study-specific hyperkalemic adverse event in 1, and study-specific non-TRACS unexplained bradycardia in 11. Three episodes carried more than one label: two were classified as both AHTR and suspected TRALI, and one as both AHTR and a hyperkalemic adverse event; therefore, event-label counts exceed the number of unique adverse-event episodes. No FNHTR or allergic/anaphylactic reaction was identified. No DHTR was identified in the available follow-up records. Among episodes with paired pre- and post-transfusion ionized calcium measurements, no post-transfusion decrease in ionized calcium was observed, and no episode reached the ionized calcium thresholds described in the TRACS criteria for citrate toxicity.

### 3.3. Univariable Associations with Transfusion-Associated Adverse Events

Results of the univariable logistic regression analyses are shown in Table 3. Pre-transfusion SIRS was associated with higher odds of the primary outcome (OR, 3.78; 95% CI, 1.36–10.51; *p* = 0.011).

None of the evaluated demographic or baseline clinical variables, including age, sex, body weight, systolic blood pressure, CRP concentration, pre-transfusion HCT, or platelet count, were significantly associated with transfusion-associated adverse events. Among transfusion-related variables, pRBC transfusion had a higher point estimate than stored whole blood, although the association did not reach statistical significance (OR, 4.12; 95% CI, 0.88–19.37; *p* = 0.073). This estimate was imprecise because only seven pRBC transfusion episodes were available. Transfusion volume, transfusion duration, and documented previous transfusion exposure were not significantly associated with transfusion-associated adverse events.

### 3.4. Patient-Clustered Multivariable Analysis

To account for multiple study-period transfusion episodes contributed by the same dog, patient-clustered GEE logistic regression was performed (Table 4). In the primary GEE model, the adjusted OR for pre-transfusion SIRS was 3.57 (95% CI, 0.98–12.98; *p* = 0.053). Although the point estimate remained elevated, the 95% CI included 1 and the association did not reach statistical significance.

pRBC transfusion also had an elevated point estimate compared with stored whole blood (adjusted OR, 3.64; 95% CI, 0.87–15.27; *p* = 0.077), although this estimate was imprecise because only seven pRBC episodes were available. Pre-transfusion platelet count was not significantly associated with transfusion-associated adverse events (adjusted OR, 0.94 per 50 K/µL increase; 95% CI, 0.86–1.03; *p* = 0.187).

In the sensitivity GEE model that additionally included documented previous transfusion exposure, the SIRS estimate remained similar (adjusted OR, 3.66; 95% CI, 0.99–13.54; *p* = 0.052). Documented previous transfusion exposure was not significantly associated with transfusion-associated adverse events (adjusted OR, 1.20; 95% CI, 0.62–2.31; *p* = 0.590).

### 3.5. Independent Re-Adjudication and Sensitivity Analyses

Independent re-adjudication of the 37 episodes originally classified as transfusion-associated adverse events classified 30 as TR present and 7 as uncertain. Imputability was graded as definite in 6 episodes, probable in 7, and possible in 24. Re-adjudicated subtype assignments were TACO in 11 episodes, TRALI in 7, AHTR in 6, combined TRALI + AHTR in 1, and other/non-TRACS events in 12.

The primary effect estimate remained directionally similar when the two fatal non-evaluable episodes were alternatively classified as events or non-events and when the original unexplained bradycardia events were excluded. Classifying both fatal episodes as transfusion-associated adverse events yielded a GEE adjusted OR of 3.87 (95% CI, 1.06–14.17; *p* = 0.041), whereas classifying both as non-events yielded an adjusted OR of 3.46 (95% CI, 0.98–12.29; *p* = 0.054). Excluding the original unexplained bradycardia events yielded an adjusted OR of 3.94 (95% CI, 0.92–16.86; *p* = 0.065).

In contrast, restricting the outcome to events assigned definite or probable imputability substantially attenuated the association (adjusted OR, 1.03; 95% CI, 0.26–4.11; *p* = 0.967). Restricting the outcome to TRs judged present by the independent reviewer yielded an adjusted OR of 2.70 (95% CI, 0.62–11.78; *p* = 0.185). Further restriction to independently judged TRs with a TRACS-defined subtype yielded an adjusted OR of 1.99 (95% CI, 0.52–7.56; *p* = 0.313) (Supplementary Table S1).

At the dog level, retaining only the first evaluable study-period transfusion episode for each of the 100 dogs yielded transfusion-associated adverse events in 22/69 SIRS dogs (31.9%) and 4/31 No SIRS dogs (12.9%), corresponding to an unadjusted OR of 3.16 (95% CI, 0.98–10.14; *p* = 0.053). Among dogs with multiple study-period transfusions, the median interval between consecutive episodes was 5.5 days (IQR, 2.25–22.25 days); repeated episodes occurred both within the same hospitalization and across subsequent admissions.

### 3.6. Number of SIRS Criteria and Transfusion-Associated Adverse Events

Primary outcome proportions were generally higher among episodes fulfilling a greater number of SIRS criteria, although the observed proportions were not strictly monotonic across individual categories. When the number of fulfilled SIRS criteria was modeled as a continuous predictor, each additional criterion was associated with a 1.63-fold increase in the odds of the primary outcome (OR, 1.63; 95% CI, 1.10–2.41; *p* = 0.014) (Table 5).

### 3.7. Non-Evaluable Fatal Episodes

Two SIRS-positive episodes were excluded from the primary outcome analysis because transfusion causality could not be reliably determined. In one episode, cardiopulmonary arrest occurred during transfusion. The dog had pre-existing hypotension and a right caudal alveolar pulmonary pattern considered consistent with pneumonia before transfusion, and no sufficiently specific new finding was available to distinguish a severe transfusion reaction from progression of the underlying disease. In the other episode, transfusion was completed approximately 4 h before cardiopulmonary arrest. Bradycardia, rigidity, nystagmus, and labored breathing were documented during transfusion, and subsequent findings included an increase in total bilirubin and pleural effusion. However, severe underlying disease and other competing causes precluded reliable attribution of the deterioration to transfusion. No necropsy was performed in either case. These two episodes were therefore retained as non-evaluable in the primary analysis and were alternatively classified as having met or not met the primary outcome in sensitivity analyses.

## 4. Discussion

In this retrospective cohort, transfusion-associated adverse events meeting the primary outcome definition were observed more frequently in transfusion episodes with pre-transfusion SIRS than in those without SIRS (32.7% vs. 11.4%). In univariable analysis, pre-transfusion SIRS was associated with higher odds of the primary outcome (OR, 3.78; 95% CI, 1.36–10.51; *p* = 0.011). In the patient-clustered GEE model, the point estimate remained elevated (adjusted OR, 3.57), although the 95% CI was wide and included 1 (0.98–12.98; *p* = 0.053). The number of fulfilled SIRS criteria was also associated with the primary outcome, with each additional criterion associated with a 1.63-fold increase in the odds of an event. However, the association was attenuated and was not statistically significant in sensitivity analyses using stricter outcome definitions based on imputability and independent re-adjudication. Accordingly, these findings should be interpreted as a hypothesis-generating association under the broad retrospective primary outcome definition rather than as evidence that pre-transfusion SIRS is an independent predictor of transfusion-associated adverse events.

Although causality cannot be inferred from the present study, the observed association is biologically plausible within the “two-hit” framework described in transfusion medicine [12]. Pre-existing systemic inflammation may represent a first inflammatory hit that promotes endothelial activation, leukocyte priming, and other pro-inflammatory responses [12,20]. Transfusion may then provide an additional stimulus through exposure to cellular components, soluble mediators, or storage-related biological changes in the transfused product [10,21,22,23]. Under this framework, dogs with pre-existing systemic inflammation could theoretically be more susceptible to additional inflammatory stimulation following transfusion. However, the present retrospective study cannot determine whether the observed association reflects a true biological susceptibility to transfusion-associated adverse events or the effects of underlying illness severity and other clinical factors.

The observed association between pre-transfusion SIRS and transfusion-associated adverse events is also difficult to interpret because many clinical features used to define SIRS overlap with those used to identify TRs [3,9]. Dogs with systemic inflammation or critical illness may already have abnormalities in temperature, heart rate, respiratory rate, blood pressure, or other clinical variables before transfusion, and these abnormalities may persist or worsen as a result of the underlying disease rather than the transfusion itself [8,13]. Primary outcome classification was performed retrospectively by a single reviewer who was not blinded to SIRS status, and formal imputability and severity grading were not part of the original assessment. Therefore, differential misclassification of primary outcome status cannot be excluded. Respiratory deterioration was particularly challenging because pneumonia, acute respiratory distress syndrome, cardiac disease, fluid administration, and progression of critical illness could mimic or coexist with TRALI or TACO [13,14,24]. For this reason, pulmonary reactions were classified as suspected TRALI or suspected TACO when definitive differentiation was not possible.

The independent re-adjudication and sensitivity analyses provided additional context for this uncertainty. When the two fatal SIRS episodes with non-evaluable primary outcome status were alternatively classified as events or non-events, the estimated association remained in the same direction. Similarly, excluding the originally identified study-specific non-TRACS unexplained bradycardia events, which do not represent a defined TR subtype under the TRACS criteria [3], did not materially change the direction of the association. In contrast, restricting the outcome to events with definite or probable imputability substantially attenuated the association toward the null. Similarly, sensitivity analyses based on independent re-adjudication yielded lower and statistically non-significant effect estimates, both when restricted to independently judged TR-present episodes and when restricted further to independently judged TR-present episodes with a TRACS-defined subtype. Although the definite/probable-imputability analysis retained only 13 events and was therefore imprecise, the attenuation observed across these stricter sensitivity analyses indicates that the estimated association was sensitive to the level of diagnostic certainty required for outcome classification and to the imputability threshold used to attribute an event to transfusion [25,26]. Importantly, independent re-adjudication was performed only for the 37 episodes meeting the primary outcome definition; the 105 episodes that did not meet the primary outcome definition were not independently re-adjudicated. Therefore, potential false-negative events among these episodes could not be assessed, and the re-adjudication should not be interpreted as validation of the overall accuracy or false-negative rate of the primary outcome classification.

All transfusion-associated adverse events meeting the primary outcome definition were acute. The most frequent event labels were suspected TACO, study-specific non-TRACS unexplained bradycardia, AHTR, and suspected TRALI, with some assigned more than one event label. No DHTR was identified in the available follow-up records. However, this finding should be interpreted cautiously because post-transfusion hospitalization duration and follow-up were not standardized. Some dogs were discharged shortly after transfusion, and subsequent assessment relied on available hospital records or documented owner communication. Delayed hemolytic reactions could therefore have been missed in dogs without adequate clinical or laboratory follow-up [3,19]. Citrate toxicity was also assessable only in episodes for which paired pre- and post-transfusion ionized calcium measurements were available [3,17]. Likewise, the lack of standardized pre- and post-transfusion thoracic imaging, oxygenation measurements, and complete hemodynamic assessment limited the ability to definitively differentiate pulmonary reaction subtypes [14,27].

Whole blood represented 95.1% of evaluable transfusion episodes, whereas only seven pRBC transfusions were included. This distribution reflected local blood-product availability rather than a systematic clinical preference for whole blood; commercial stored whole blood was readily available, whereas pRBCs were prepared in-house from donated whole blood when required. Although pRBC transfusion showed an elevated point estimate in both univariable and GEE analyses, the confidence intervals were wide and the small number of pRBC episodes precluded a meaningful comparison of primary outcome occurrence between product types. These findings should therefore not be interpreted as evidence that pRBCs are associated with a higher frequency of transfusion-associated adverse events than stored whole blood. The predominance of whole blood also limits generalizability to hospitals in which component therapy is more commonly used.

Previous and repeated transfusion exposure also varied among dogs and may have influenced primary outcome occurrence. Documented previous transfusion exposure was recorded as a binary history and included transfusions identified from available hospital or referral records or owner-reported history; therefore, the exact lifetime number of prior transfusions could not be reliably determined for all dogs. In addition, dogs could contribute multiple study-period transfusion episodes, occurring either during the same hospitalization or at subsequent admissions. Because pre-transfusion SIRS status could vary between episodes within the same dog, the transfusion episode was retained as the primary unit of analysis, with within-dog correlation accounted for using GEE. The first-evaluable-episode-only analysis produced an effect estimate in the same direction as the primary analysis, although the 95% CI included 1. Donor identity and the number of distinct donor exposures could not be consistently reconstructed, preventing assessment of donor-specific exposure or potential alloimmunization-related effects [28].

Baseline differences between groups also suggest the possibility of residual confounding. Dogs in the SIRS group had lower SBP and HCT, with trends toward higher CRP concentrations and lower platelet counts. These differences may indicate that pre-transfusion SIRS partly reflects greater overall illness severity rather than a specific susceptibility to transfusion-associated adverse events. The study population included a wide range of underlying diseases, and many dogs had multiple concurrent clinical problems. Although the overall distribution of disease categories did not differ significantly between groups, the limited number of primary outcome events and the small numbers within several disease categories restricted our ability to account for underlying disease, disease severity, hemorrhage, immune-mediated disease, sepsis, fluid therapy, and other features of critical illness in the multivariable analysis. Thus, residual confounding cannot be excluded and should be considered when interpreting the observed association between pre-transfusion SIRS and transfusion-associated adverse events.

The SIRS exposure itself also had limitations inherent to retrospective classification. Temperature, heart rate, and respiratory rate were based on values recorded immediately before transfusion, and a single measurement may be influenced by transient pain, stress, excitement, panting, or other non-inflammatory factors. Respiratory rate was analyzed as recorded and was not overridden when panting, pain, or stress was suspected, potentially reducing the specificity of the respiratory SIRS criterion [8,29]. In addition, quantitative band-neutrophil percentages were not consistently available, and the leukocyte criterion was therefore based on total WBC count. These factors may have resulted in misclassification of pre-transfusion SIRS status [8].

Other limitations include the retrospective, single-center design, the relatively small number of primary outcome events, and the resulting limited precision of multivariable estimates. Exact storage age could not be reconstructed for every blood unit, and donor identity could not be consistently linked across transfusion episodes. All dogs received transfusion premedication according to the hospital protocol, which prevents evaluation of the influence of premedication on primary outcome occurrence and may limit generalizability to settings in which premedication is not routinely used [6,17]. Post-transfusion monitoring was individualized rather than prospectively standardized. Given the limited number of primary outcome events and the heterogeneity and overlap of underlying diseases, more extensive adjustment for disease-related variables would have increased model complexity and the risk of overfitting and unstable estimates. These limitations should be considered when interpreting the observed frequency of transfusion-associated adverse events and the association between pre-transfusion SIRS and transfusion-associated adverse events.

Despite these limitations, pre-transfusion SIRS was associated with a higher observed frequency of transfusion-associated adverse events under the broad retrospective primary outcome definition, and a greater number of fulfilled SIRS criteria was associated with increased odds of the primary outcome when modeled continuously. Given the attenuation observed under stricter outcome definitions, these findings should be considered hypothesis-generating. Prospective multicenter studies using standardized baseline measurements, predefined reaction criteria [30], systematic post-transfusion follow-up, and blinded adjudication of all transfusion episodes are needed to determine whether systemic inflammatory status is independently associated with transfusion-associated adverse events.

## 5. Conclusions

Pre-transfusion SIRS was associated with a higher observed frequency of transfusion-associated adverse events under the broad retrospective primary outcome definition, and a greater number of fulfilled SIRS criteria was associated with higher odds of the primary outcome when modeled continuously. However, the association was attenuated and was not statistically significant in sensitivity analyses using stricter outcome definitions based on imputability and independent re-adjudication. These findings should therefore be considered hypothesis-generating. Prospective multicenter studies using standardized outcome definitions and blinded adjudication of all transfusion episodes are needed to clarify whether systemic inflammatory status is independently associated with transfusion-associated adverse events.

## Figures and Tables

**Table 1:** Baseline characteristics of SIRS-classifiable transfusion episodes according to pre-transfusion SIRS status.

Characteristic	Overall (*N* = 145)	No SIRS (*n* = 45)	SIRS (*n* = 100)	*p*-Value
Demographic and baseline clinical variables				
Age, years	11.58 [8.58–13.33]	11.83 [8.33–13.67]	11.46 [9.42–13.02]	0.463
Sex				
Male	90 (62.1%)	28 (62.2%)	62 (62.0%)	1.000
Female	55 (37.9%)	17 (37.8%)	38 (38.0%)	
Body weight, kg	4.45 [3.23–6.60]	4.13 [3.06–6.26]	4.88 [3.35–8.76]	0.190
Systolic blood pressure, mmHg	120.0 [110.0–140.0]	130.0 [120.0–150.0]	120.0 [107.2–140.0]	0.006
C-reactive protein	67.16 [30.10–97.76]	47.63 [26.55–78.60]	73.20 [36.37–101.75]	0.052
Pre-transfusion HCT, %	17.3 [14.7–20.8]	17.7 [15.2–22.9]	16.7 [14.6–20.0]	0.034
Pre-transfusion platelet count, K/µL	109.0 [40.0–221.0]	157.0 [59.0–321.0]	89.5 [35.5–203.2]	0.051
SIRS-defining variables				
Temperature, °C	38.3 [38.0–38.7]	38.3 [38.1–38.6]	38.3 [37.8–38.7]	
Heart rate, beats/min	120 [108–144]	120 [108–120]	138 [114–150]	
Respiratory rate, breaths/min	24 [18–30]	18 [18–18]	24 [18–36]	
WBC, K/µL	18.05 [11.10–27.47]	12.30 [9.71–15.59]	21.57 [14.47–32.84]	
Underlying disease category				
Immune-mediated	12 (8.3%)	1 (2.2%)	11 (11.0%)	0.067
Infectious/Inflammatory	3 (2.1%)	0 (0.0%)	3 (3.0%)	
Neoplasia	31 (21.4%)	7 (15.6%)	24 (24.0%)	
Gastrointestinal	18 (12.4%)	7 (15.6%)	11 (11.0%)	
Hepatobiliary	9 (6.2%)	4 (8.9%)	5 (5.0%)	
Renal	7 (4.8%)	5 (11.1%)	2 (2.0%)	
Hemorrhage	7 (4.8%)	2 (4.4%)	5 (5.0%)	
Anemia-other	4 (2.8%)	3 (6.7%)	1 (1.0%)	
Endocrine/Metabolic	1 (0.7%)	0 (0.0%)	1 (1.0%)	
Other	53 (36.6%)	16 (35.6%)	37 (37.0%)	

Data are presented as median [interquartile range] for continuous variables and number (percentage) for categorical variables, unless otherwise indicated. Continuous variables were compared using the Mann–Whitney U test, and categorical variables were compared using the chi-square test or Fisher’s exact test, as appropriate. CRP was available for 90 of 145 episodes (No SIRS, *n* = 27; SIRS, *n* = 63); all other variables shown were available for all 145 SIRS-classifiable episodes. SIRS-defining variables (temperature, heart rate, respiratory rate, and WBC count) were summarized descriptively and were not subjected to between-group hypothesis testing because these variables were used to define SIRS status. Because several cells in the underlying disease-category analysis had small expected counts, the global chi-square result should be interpreted cautiously. SIRS, systemic inflammatory response syndrome; HCT, hematocrit; CRP, C-reactive protein.

**Table 2:** Transfusion characteristics and occurrence of transfusion-associated adverse events according to pre-transfusion SIRS status.

Characteristic	Overall (*N* = 142)	No SIRS (*n* = 44)	SIRS (*n* = 98)	*p*-Value
Blood product				
Stored whole blood	135 (95.1%)	42 (95.5%)	93 (94.9%)	0.887
pRBC	7 (4.9%)	2 (4.5%)	5 (5.1%)	
Transfusion volume, mL/kg	22.9 [18.7–28.1]	23.4 [19.2–26.1]	22.6 [18.2–28.3]	0.949
Transfusion duration, h	7.0 [6.0–9.0]	7.0 [6.0–10.0]	8.0 [6.0–9.0]	0.744
Documented previous transfusion exposure	47 (33.1%)	15 (34.1%)	32 (32.7%)	1.000
Any transfusion-associated adverse event	37 (26.1%)	5 (11.4%)	32 (32.7%)	0.007

Data are presented as median [interquartile range] for continuous variables and number (percentage) for categorical variables, unless otherwise indicated. All 37 identified transfusion-associated adverse events were acute. Documented previous transfusion exposure was defined as at least one prior transfusion documented in available hospital or referral records or in the owner-reported medical history, including transfusions occurring before the study period. SIRS, systemic inflammatory response syndrome; pRBC, packed red blood cells.

**Table 3:** Univariable logistic regression analysis for occurrence of any transfusion-associated adverse event.

Predictor	*n*	Unadjusted OR	95% CI	*p*-Value	Reference/Unit
**Primary exposure**					
Pre-transfusion SIRS	142	3.78	1.36–10.51	0.011	No SIRS
**Demographic/baseline** **clinical** **variables**					
Age	142	1.02	0.92–1.13	0.736	per 1 year
Male sex	142	1.33	0.60–2.95	0.475	Female
Body weight	142	1.00	0.95–1.05	0.917	per 1 kg
Systolic blood pressure	142	1.01	0.88–1.16	0.900	per 10 mmHg
CRP	88	1.01	0.92–1.11	0.822	per 10 mg/L
Pre-transfusion HCT	142	0.85	0.57–1.27	0.438	per 5 percentage points
Pre-transfusion platelet count	142	0.91	0.81–1.03	0.149	per 50 K/µL
**Transfusion** **-related** **variables**					
pRBC transfusion	142	4.12	0.88–19.37	0.073	Stored whole blood
Transfusion volume	142	0.95	0.76–1.20	0.691	per 5 mL/kg
Transfusion duration	142	0.99	0.87–1.13	0.926	per 1 h
Documented previous transfusion exposure	142	1.33	0.61–2.90	0.477	No previous exposure

Univariable logistic regression was performed using the primary outcome as the dependent variable. Analyses were restricted to 142 transfusion episodes with evaluable primary outcomes. CRP was available for 88 of 142 episodes; all other predictors shown were available for all 142 episodes. Odds ratios for continuous variables are expressed according to the increments specified in the “Reference/unit” column. OR, odds ratio; CI, confidence interval; SIRS, systemic inflammatory response syndrome; CRP, C-reactive protein; HCT, hematocrit; pRBC, packed red blood cells.

**Table 4:** Patient-clustered multivariable generalized estimating equation models for occurrence of any transfusion-associated adverse event.

Model/Predictor	Adjusted OR	95% CI	*p*-Value	Reference/Unit
**Primary GEE model**				
Pre-transfusion SIRS	3.57	0.98–12.98	0.053	No SIRS
pRBC transfusion	3.64	0.87–15.27	0.077	Stored whole blood
Pre-transfusion platelet count	0.94	0.86–1.03	0.187	per 50 K/µL
**Sensitivity GEE model**				
Pre-transfusion SIRS	3.66	0.99–13.54	0.052	No SIRS
pRBC transfusion	3.52	0.79–15.70	0.099	Stored whole blood
Pre-transfusion platelet count	0.94	0.86–1.03	0.198	per 50 K/µL
Documented previous transfusion exposure	1.20	0.62–2.31	0.590	No previous exposure

Generalized estimating equation (GEE) logistic regression was used to account for within-dog correlation arising from multiple study-period transfusion episodes contributed by the same dog. The sensitivity model additionally included documented previous transfusion exposure. OR, odds ratio; CI, confidence interval; GEE, generalized estimating equation; SIRS, systemic inflammatory response syndrome; pRBC, packed red blood cells.

**Table 5:** Transfusion-associated adverse events according to the number of fulfilled SIRS criteria.

Number of SIRS Criteria	Transfusion Episodes, *n*	Transfusion-Associated Adverse Events, *n*	Primary Outcome Proportion, %	95% CI
0	9	0	0.0	0.0–29.9
1	35	5	14.3	6.3–29.4
2	49	17	34.7	22.9–48.7
3	39	10	25.6	14.6–41.1
4	10	5	50.0	23.7–76.3
**Overall**	**142**	**37**	**26.1**	**19.** **5** **–** **33.8**

Primary outcome proportions are presented as the number and percentage of transfusion episodes with a transfusion-associated adverse event within each SIRS-criterion category. The 95% confidence intervals for primary outcome proportions were calculated using the Wilson score method. When the number of fulfilled SIRS criteria was modeled as a continuous predictor, each additional criterion was associated with a 1.63-fold increase in the odds of the primary outcome (OR, 1.63; 95% CI, 1.10–2.41; *p* = 0.014). SIRS, systemic inflammatory response syndrome; OR, odds ratio; CI, confidence interval.

## Data Availability

The data supporting the findings of this study are available from the corresponding author upon reasonable request. The data are not publicly available because they were obtained retrospectively from the medical records of client-owned dogs and may contain information subject to confidentiality considerations.

## Supplementary Materials

The following supporting information can be downloaded at https://www.mdpi.com/article/10.3390/ani16203287/s1: Table S1: Sensitivity analyses for the association between pre-transfusion SIRS and transfusion-associated adverse events.

## Abbreviations

The following abbreviations are used in this manuscript:

AHTR	Acute hemolytic transfusion reaction
CBC	Complete blood count
CI	Confidence interval
CPDA	Citrate-phosphate-dextrose-adenine
CRP	C-reactive protein
DEA	Dog erythrocyte antigen
DHTR	Delayed hemolytic transfusion reaction
FNHTR	Febrile non-hemolytic transfusion reaction
GEE	Generalized estimating equation
HCT	Hematocrit
IQR	Interquartile range
OR	Odds ratio
pRBC	Packed red blood cells
SBP	Systolic blood pressure
SIRS	Systemic inflammatory response syndrome
TACO	Transfusion-associated circulatory overload
TR	Transfusion reaction
TRACS	Transfusion Reaction Small Animal Consensus Statement
TRALI	Transfusion-related acute lung injury
WBC	White blood cell count
