# Supplementary material for: Association Between Pre-Transfusion Systemic Inflammatory Response Syndrome and Transfusion-Associated Adverse Events in Dogs: A Retrospective Study
Source: Animals (Basel). 2026 Oct 18;16(20):3287. doi: 10.3390/ani16203287 (PMC13700586; doi:10.3390/ani16203287)
Supplement: Supplementary file 1 [file animals-16-03287-s001.zip › animals-4534561-supplementary.pdf]

Supplementary Table S1. Sensitivity analyses for the association between pre-transfusion SIRS and transfusion-associated adverse events.

Sensitivity scenario	SIRS	No SIRS	Unadjusted OR (95% CI)	*p*-Value	GEE adjusted OR (95% CI)	*p*-Value
Primary analysis	32/98 (32.7%)	5/44 (11.4%)	3.78 (1.36–10.51)	0.011	3.57 (0.98–12.98)	0.053
Both fatal episodes classified as meeting the primary outcome	34/100 (34.0%)	5/44 (11.4%)	4.02 (1.45–11.13)	0.007	3.87 (1.06–14.17)	0.041
Both fatal episodes classified as not meeting the primary outcome	32/100 (32.0%)	5/44 (11.4%)	3.67 (1.32–10.19)	0.013	3.46 (0.98–12.29)	0.054
Excluding study-specific non-TRACS unexplained bradycardia events	23/98 (23.5%)	3/44 (6.8%)	4.19 (1.19–14.80)	0.026	3.94 (0.92–16.86)	0.065
Definite/probable transfusion imputability only	10/98 (10.2%)	3/44 (6.8%)	1.55 (0.41–5.95)	0.520	1.03 (0.26–4.11)	0.967
Independent re-adjudication: reaction present	25/98 (25.5%)	5/44 (11.4%)	2.67 (0.95–7.53)	0.063	2.70 (0.62–11.78)	0.185
Independent re-adjudication: reaction present with a TRACS-defined subtype	15/98 (15.3%)	3/44 (6.8%)	2.47 (0.68–9.02)	0.171	1.99 (0.52–7.56)	0.313

SIRS, systemic inflammatory response syndrome; OR, odds ratio; CI, confidence interval; GEE, generalized estimating equation; TRACS, Transfusion Reaction Small Animal Consensus Statement. Independent re-adjudication was performed only on the 37 episodes meeting the primary outcome definition; the 105 episodes that did not meet the primary outcome definition were not independently re-adjudicated. Therefore, the re-adjudication-based sensitivity analyses assessed the diagnostic certainty and robustness of the originally identified primary outcome events but could not evaluate potential false-negative events among episodes that did not meet the primary outcome definition. Accordingly, the re-adjudication should not be interpreted as validation of the overall accuracy or false-negative rate of the primary outcome classification. In the re-adjudication-based sensitivity analyses, "reaction present" refers to the independent reviewer's retrospective classification and does not necessarily indicate a definitively confirmed transfusion reaction.
